# A Yeast Two-Hybrid Screen for SYP-3 Interactors Identifies SYP-4, a Component Required for Synaptonemal Complex Assembly and Chiasma Formation in *Caenorhabditis elegans* Meiosis

**DOI:** 10.1371/journal.pgen.1000669

**Published:** 2009-10-02

**Authors:** Sarit Smolikov, Kristina Schild-Prüfert, Mónica P. Colaiácovo

**Affiliations:** Department of Genetics, Harvard Medical School, Boston, Massachusetts, United States of America; Institut Jean-Pierre Bourgin, INRA de Versailles, France

## Abstract

The proper assembly of the synaptonemal complex (SC) between homologs is critical to ensure accurate meiotic chromosome segregation. The SC is a meiotic tripartite structure present from yeast to humans, comprised of proteins assembled along the axes of the chromosomes and central region (CR) proteins that bridge the two chromosome axes. Here we identify SYP-4 as a novel structural component of the SC in *Caenorhabditis elegans*. SYP-4 interacts in a yeast two-hybrid assay with SYP-3, one of components of the CR of the SC, and is localized at the interface between homologs during meiosis. SYP-4 is essential for the localization of SYP-1, SYP-2, and SYP-3 CR proteins onto chromosomes, thereby playing a crucial role in the stabilization of pairing interactions between homologous chromosomes. In the absence of SYP-4, the levels of recombination intermediates, as indicated by RAD-51 foci, are elevated in mid-prophase nuclei, and crossover recombination events are significantly reduced. The lack of chiasmata observed in *syp-4* mutants supports the elevated levels of chromosome nondisjunction manifested in high embryonic lethality. Altogether our findings place SYP-4 as a central player in SC formation and broaden our understanding of the structure of the SC and its assembly.

## Author Summary

Meiosis is a two-part cell division program that ensures the formation of haploid gametes (e.g. eggs and sperm), which can then reconstitute a species' ploidy through fertilization. A critical step towards accomplishing this task is the accurate segregation of homologous chromosomes away from each other during meiosis I. This requires the formation of at least one obligatory crossover event (genetic exchange) between each pair of homologous chromosomes. In most organisms, the formation of all crossover events greatly relies on the synaptonemal complex (SC). This “zipper-like” structure holds the pairs of homologous chromosomes together during meiotic prophase I, and crossover recombination is completed in the context of the fully formed SCs. Here, we identify SYP-4 as a novel structural component of the SC in the nematode *C. elegans*. In its absence, SCs fail to form, resulting in a lack of crossover formation and increased errors in chromosome segregation. SYP-4 interacts in a yeast two-hybrid assay with SYP-3, one of the SC proteins, and its localization onto chromosomes is interdependent with SYP-1, SYP-2, and SYP-3 proteins. SYP-4 therefore plays a critical role during *C. elegans* meiosis in generating the ultrastructurally conserved SC that is ubiquitously present from yeast to humans.

## Introduction

The synaptonemal complex (SC) is a proteinaceous structure formed between each pair of homologous chromosomes during meiotic prophase I. Meiotic recombination unfolds, and crossover events are completed, in the context of this structure. The formation of these crossover events is a prerequisite for accurate chromosome segregation at the first meiotic division since it ensures that homologous chromosomes will be held together until anaphase I. Subsequently, sister chromatids separate in the second meiotic division resulting in the formation of haploid gametes. Without a functional SC these events are impaired, resulting either in a meiotic arrest or in increased chromosome nondisjunction.

The SC is composed of two main parts: the axes and the central region. The axes-associated proteins assemble along the chromosomes, followed by the central region proteins, which proceed to connect homologous chromosomes axes thereby completing synapsis. This temporal separation in the assembly of these two SC sub-structures is supported by the observation made in various organisms that the lack of axis assembly results in severe defects in the recruitment of central region proteins [1]–[3].

The central region of the SC is comprised of proteins that form transverse filaments (TF) connecting or bridging homologous chromosomes. These TF proteins share a common structure: a central coiled-coil domain flanked by globular domains [4],[5]. However, central region components notoriously lack a significant level of sequence conservation throughout species, which has hindered sequence-based efforts in identifying novel components. Remarkably though, the SC is highly conserved at the ultrastructural level. Specifically, studies in mice, flies and yeast suggest that the central region of the SC is comprised of pairs of central region proteins arranged in a head-to-head orientation with their N-termini positioned at the center of the SC [6]–[8]. Therefore, the assembly of multiple units along the chromosomes results in a zipper-like organization. Since coiled-coil domains show a propensity to dimerize it has been proposed that central region proteins form parallel dimers through their coiled-coil domains [7],[9]. Studies of the yeast TF protein Zip1 revealed dimers and high-order multimers forming *in vitro* [8], further supporting this model. Detailed deletion studies in yeast revealed that TFs span the distance between homologous chromosomes, specifically demonstrating that the coiled-coil domain is the main determinant of the width of the SC [9]. Zip1 is the only TF protein reported thus far for yeast and is therefore the sole component of the central region in this organism [10]. Flies have two central region proteins: C(3)G and CONA, but only C(3)G was proposed to act as the *Drosophila* TF protein [11],[12]. In *C. elegans*, three central region proteins have already been identified: SYP-1, SYP-2, and SYP-3, but due to their relatively small size it is reasonable to speculate that these proteins act cooperatively as TFs [2],[13],[14].

Electron microscopy (EM) analyses performed in various model systems revealed an electron-dense linear structure, called the central element, located along the middle of the central region of the SC [4]. Until recently, it was unclear whether this structure resulted from the overlap between the globular domains of the central region proteins, or was formed by central element-specific proteins localizing only at the middle of the SC. However, studies performed in mice revealed the identity of several proteins specifically localizing to the central element and identified their function in promoting the assembly of the SC as a 3-dimensional structure [15],[16]. Central element proteins may therefore not only act as “clamps” holding the transverse filaments of the SC, but may also have a role in promoting SC assembly in a particular direction, determining its height (SYCE1) [16],[17] or length (SYCE2 and TEX12) [15],[18]. Although a central element has not yet been observed by EM analysis in *C. elegans*, it is possible that some of the central region proteins in this system perform a similar function to central element proteins from mice (K.P.S. and M.P.C. unpublished data).

The formation of the SC is tightly coordinated with the progression of recombination. The SC starts to form at the entry to meiotic prophase I during leptotene. The first meiotic DNA double-strand breaks (DSBs) are observed at this stage, and in some organisms, such as in yeast, plants and mice, DSB formation is essential for SC assembly [19],[20]. In contrast, both in *D. melanogaster* and *C. elegans*, recombination is dispensable for SC formation [21],[22]. Throughout these various organisms, the mature SC is observed at the pachytene stage where crossover recombination is completed. Deletion of genes encoding SC central region proteins results in lack of synapsis and defects in the progression of recombination. In yeast mutants lacking the central region protein Zip1, crossovers are reduced to 25% of the levels observed in wild-type [23], while male mice lacking SYCP1 proteins exhibit a pachytene arrest accompanied by an accumulation of mid to late recombination markers [24]. In *D. melanogaster* and *C. elegans*, synapsis is also crucial for recombination, given that crossover formation is impaired and chiasmata are not observed in SC-deficient mutants [2], [12]–[14].

To further investigate the structure of the SC in *C. elegans*, we performed a yeast two-hybrid screen utilizing the known SC central region proteins as baits. This has resulted in the identification of SYP-4, a novel component of the central region of the SC, and the first example of the identification of a SC structural protein through the yeast two-hybrid approach. Here, we show that SYP-4 displays all the hallmark features of an SC component. Specifically, SYP-4 is essential for chromosome synapsis, and in its absence, chromosomes initiate pairing interactions that cannot be stabilized. These defects result in increased germ cell apoptosis due to impaired DSB repair progression. In addition, crossover frequencies are severely reduced in *syp-4* mutants, resulting in increased chromosome nondisjuction. SYP-4 localizes at the interface between homologous chromosomes during meiosis and this localization requires axis morphogenesis and is interdependent with the SYP-1, SYP-2 and SYP-3 proteins. Moreover, SYP-4 interacts with SYP-3 in a yeast two-hybrid assay, suggesting that the function of SYP-4 is executed through its role as a member of the CR of the SC. Our discovery of SYP-4 therefore sheds new light on the structure of the SC in *C. elegans* and the roles its proteins play in meiosis.

## Results

### SYP-4 Is Essential for Chromosome Synapsis and Chiasma Formation during *C. elegans* Meiosis

**Figure 1:**
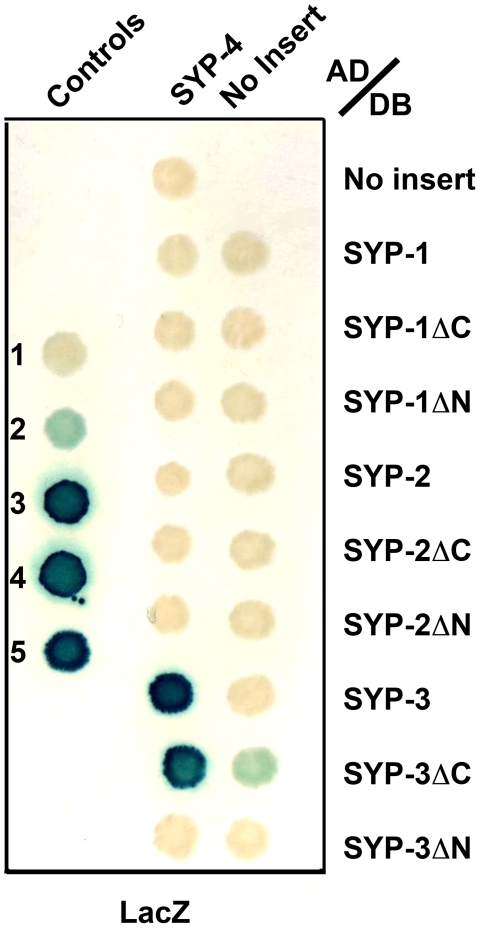
A yeast two-hybrid approach reveals that SYP-4 interacts with SYP-3 through its N-terminal domain. The yeast two-hybrid system was used to test for protein interactions between SYP-1, SYP-2 and SYP-3 full length, N- (ΔN) and C-terminal (ΔC) truncations fused to the DNA binding domain (DB) of GAL4, and SYP-4 full length fused to the activation domain (AD) of GAL4. Positive yeast two-hybrid interactions were assessed by β-galactosidase activity. This approach revealed an interaction between SYP-4 and SYP-3. Numbers 1 to 5 represent standard controls: 1, DB and AD without any fusion; 2, DB-pRB and AD-E2F1; 3, DB-Fos and AD-Jun; 4, Gal4p and AD; and 5, DB-DP and AD-E2F1.

**Figure 2:**
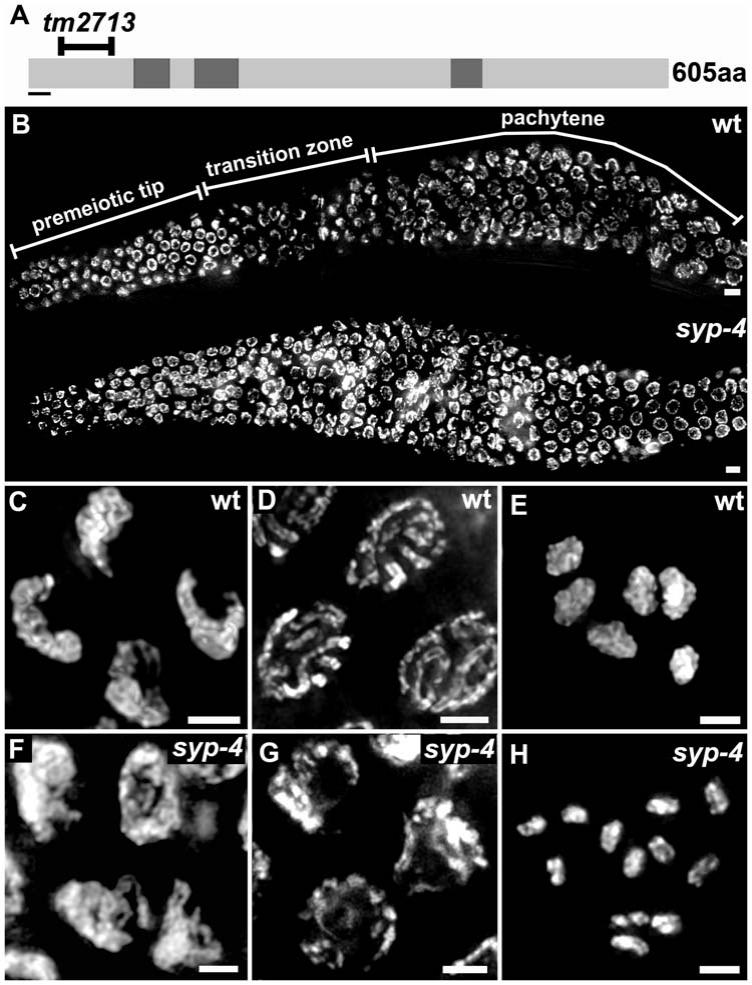
Extended transition zone chromosome morphology and lack of chiasmata in meiotic prophase I nuclei in *syp-4* mutants. (A) Schematic representation of the predicted SYP-4 protein. The region deleted in the *tm2713* mutant allele is indicated (codons 28 through 84). Coiled-coil domains, with start and end points identified through the COILS program are shaded in dark gray. The black bar indicates the N-terminal region used for antibody production. (B) Low magnification images of DAPI-stained nuclei of whole-mount gonads from age-matched wild type and *syp-4(tm2713)* adult hermaphrodites. The extended transition zone chromosome configuration is observed until late pachytene in the latter. Progression from early to mid-prophase is observed from left to right. (C–H) High magnification images of DAPI-stained nuclei at transition zone (C,F), pachytene (D,G), and diakinesis (E,G) in wild type (C–E) and *syp-4(tm2713)* mutants (F–H). Bars, 5 µm (B) and 2 µm (C–H).

We applied a yeast two-hybrid approach to identify novel proteins functioning in the SC in *C. elegans*. Specifically, we screened a cDNA library prepared from mixed-stage worms, utilizing SYP-1, SYP-2 and SYP-3 full-length constructs as well as N- and C-terminal truncations as baits (see Materials and Methods). As a result, we identified SYP-4 (encoded by open reading frame H27M09.3) as a protein that interacts with both the full-length and C-terminal truncation constructs of SYP-3. Screens performed with SYP-1 and SYP-2 as baits failed to identify SYP-4 as an interacting protein. In addition, when directly tested for a yeast two-hybrid interaction, SYP-4 failed to interact with the full length, as well as the C- or N-terminal truncation constructs of SYP-1 and SYP-2 (Figure 1, Figure S1). SYP-4 encodes for a 605 amino acid protein. It is predicted to contain three stretches of coiled-coil structure in the region between residues 115 and 410, based on analysis using the COILS program [25]. SYP-4 lacks any other evident structural domains or shared homology with other proteins in *C. elegans* or other organisms (Figure 2A).

To further investigate the role of SYP-4 in meiosis we examined the phenotype of *syp-4(tm2713)* mutants. These mutants carry a 213 bp out-of-frame deletion in the N-terminus of *syp-4* (Figure 2A), predicted to result in the absence of a fully functional SYP-4 protein in these worms. In addition, genetic analysis indicates that *tm2713* is a null allele of *syp-4* (see Materials and Methods). *syp-4(tm2713)* mutants exhibit high levels of embryonic lethality (97.5%, n = 1855) and a high percentage of males (40%) among their surviving progeny compared to wild type (0% and 0.2%, respectively, n = 1798), which are phenotypes suggestive of errors in meiotic chromosome segregation.

Analysis of chromosome morphogenesis in *syp-4(tm2713)* mutant gonads revealed defects in the progression of meiotic prophase I. As observed in wild type, chromosomes clustered towards one side of the nuclei upon entering into prophase I in *syp-4(tm2713)* mutants, therefore acquiring the polarized configuration that is characteristic of transition zone (leptotene/zygotene) nuclei (Figure 2B, 2C, and 2F). However, in contrast to wild type, chromosomes failed to redisperse throughout the nuclear periphery upon entrance into pachytene in *syp-4(tm2713)* mutants. Instead, they remained mostly clustered until late pachytene in an “extended transition zone” morphology characteristic of null mutants for genes encoding proteins that constitute the central region of the SC [2],[13],[14] (Figure 2B, 2D, and 2G). Moreover, when chromosomes redispersed in late pachytene nuclei in *syp-4(tm2713)* mutants, the thick parallel DAPI-stained tracks indicative of synapsed chromosomes observed in wild type were not apparent, and instead, thin DAPI-stained tracks were present suggesting defects in synapsis. In addition, transmission electron microscopy (TEM) analysis revealed a lack of SC formation in *syp-4(tm2713)* mutants, suggesting that the defects in synapsis stem from an inability to form the SC structure in the absence of SYP-4 (Figure S2). As nuclei progressed into diakinesis, a complete lack of chiasmata was observed. Therefore, instead of the 6 DAPI-stained bodies present in wild type diakinesis oocytes, corresponding to the six pairs of attached homologous chromosomes, 11.9 DAPI-stained bodies (n = 31) were observed in *syp-4* mutants (Figure 2E and 2H). Altogether, this analysis implicates SYP-4 in playing a crucial role in chromosome synapsis and chiasma formation.

### SYP-4 Localizes to Chromosomes in a SYP-1–, SYP-2–, and SYP-3–Dependent Manner

**Figure 3:**
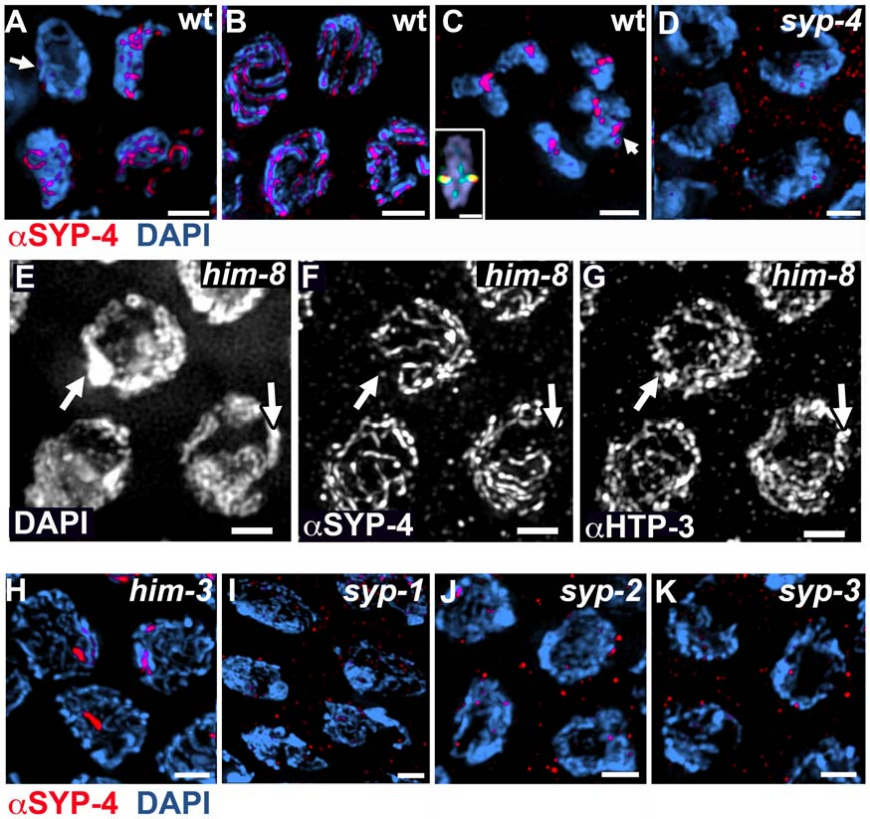
SYP-4 localizes to chromosomes during prophase I, and its localization requires both axis-associated and CR proteins. (A–C) High magnification images of nuclei stained with DAPI and anti-SYP-4. Wild type nuclei at transition zone (A), late pachytene (B), and diakinesis (C), showing immunolocalization of SYP-4 on chromosomes throughout meiotic prophase I. Arrow in (A) indicates a premeiotic nucleus adjacent to the meiotic transition zone nuclei. Arrow in (C) indicates two superimposed bivalents. Inset in (C) indicates a single bivalent at high magnification, co-immunostained with SYP-4 (red) and HTP-3 (green). SYP-4 localizes to the region distal to the chiasma (short arms of the bivalent). (D) Pachytene nuclei exemplifying the lack of SYP-4 immunolocalization on chromosomes observed in *syp-4* mutants. (E–G) SYP-4 is excluded only from unsynapsed chromosomes (arrows) observed in *him-8* mutants. (H–K) SYP-4 localization in pachytene nuclei is perturbed in *him-3* (H), *syp-1* (I), *syp-2* (J), and *syp-3* (K) mutants, indicating that SYP-4 localization requires both normal axis-morphogenesis and the presence of central region proteins. Bars, 2 µm.

To examine the immunolocalization of SYP-4 on whole mounted germlines, an α-SYP-4 antibody was raised against the N-terminus (first 27 amino acids) of SYP-4. The specificity of this antibody was confirmed by detecting the presence of SYP-4 signal on meiotic chromosomes in wild type nuclei (Figure 3A–3C) and not in *syp-4(tm2713)* mutants (Figure 3D). In wild type, SYP-4 was first detected upon entrance into meiosis as foci or short tracks on chromosomes in transition zone nuclei (Figure 3A). SYP-4 remained associated with chromosomes throughout pachytene where it was observed between synapsed chromosomes (Figure 3B) [2],[13],[14],[26],[27]. During the transition from late pachytene into diplotene, the SC starts to disassemble and chromosome remodeling unfolds around the crossover site [28]–[30]. At this transition, SYP-4 signal was greatly reduced throughout most of the length of the chromosomes, becoming mostly concentrated towards one end of each chromosome. This asymmetric localization pattern is similar to that observed for the other SYP proteins during this transition [28]. Given that a single crossover (obligate crossover) is formed between each pair of homologous chromosomes in *C. elegans* [31] and this crossover is off-center, chromosome remodeling then results in bivalents at diakinesis with a cross-shaped configuration composed of a long and a short axes intersecting at the chiasma [28]. By early diakinesis, SYP-4 localization was restricted to the short axes (short arms; the region distal to the chiasma) (Figure 3C), and by the end of diakinesis it was no longer detectable on chromosomes. This distinct pattern of localization is unique to SC central region proteins, as lateral element proteins remain on both arms of the bivalent through the end of diakinesis [2],[13],[14],[26],[27].

To further examine if SYP-4 acts as a central region protein, we tested whether SYP-4 localizes to unsynapsed chromosomes in a scenario in which lateral element assembly is normal, but the central region is not formed. In *him-8* mutants, all autosomes are synapsed and exhibit normal localization of central region proteins, while the X chromosome remains unsynapsed and shows no localization of central region proteins despite normal axis morphogenesis [32]. We did not detect any SYP-4 association with the unsynapsed pair of chromosomes in the *him-8* mutants (Figure 3E–3G). These results further support a role for SYP-4 as a central region protein of the SC.

We next examined the requirements for SYP-4 localization. First, we determined whether SYP-4 localization is dependent on the lateral element protein HIM-3 [27] and on the SC central region proteins SYP-1, SYP-2 and SYP-3 [2],[13],[14]. In the absence of HIM-3, SYP-4 was observed only as a dot or short patch associated with chromosomes in pachytene nuclei (Figure 3H), similarly to the localization of SYP-1, SYP-2 and SYP-3 in *him-3(RNAi)* or *him-3* null mutants [2],[13],[14] [33]. This suggests that SYP-4 depends on normal axis morphogenesis for its assembly onto chromosomes. Central region proteins were also essential for SYP-4 localization, given that SYP-4 localization was not observed in *syp-1*, *syp-2* or *syp-3* null mutants (Figure 3I–3K).

**Figure 4:**
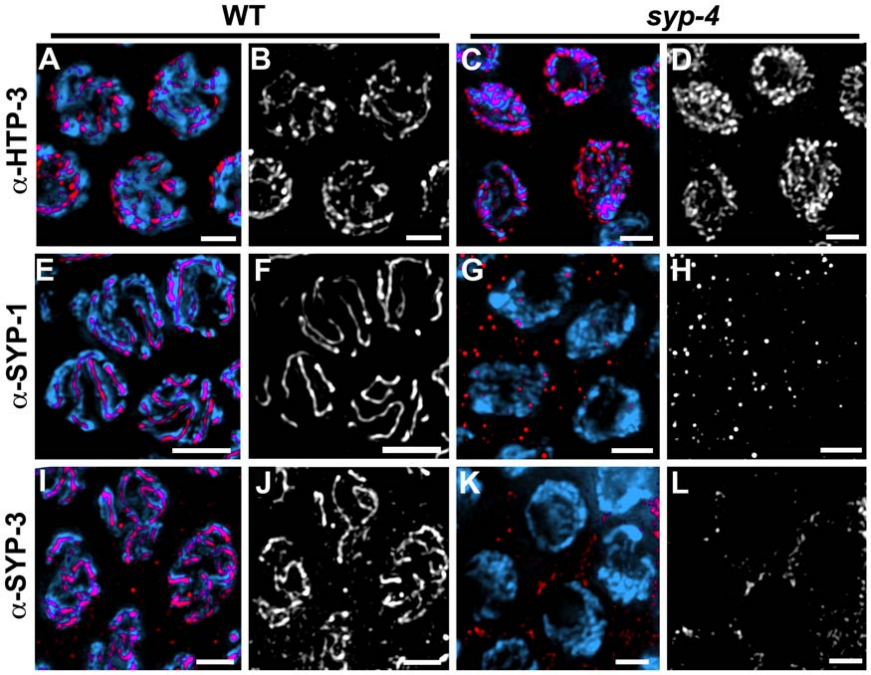
Central region proteins, but not axis-associated proteins, localize to chromosomes in a SYP-4-dependent manner. High magnification images of pachytene nuclei in wild type (A,B,E,F,I,J) and *syp-4* mutants (C,D,G,H,K,L). DAPI-stained chromosomes are immunostained with anti-HTP-3 to observe the lateral elements (A–D), and anti-SYP-1 (E–H) or anti-SYP-3 (I–L) to visualize the central region of the SC. In *syp-4* mutants, HTP-3 is observed localizing continuously along chromosomes (C,D), indicating that lateral element formation is normal. In contrast, SYP-1 and SYP-3 signals are not observed on chromosomes in *syp-4* mutants (G,H,K,L), indicating lack of central region formation. Bars, 2 µm.

In contrast, the localization of axis-associated proteins, such as HIM-3 [27] or HTP-3 [34], was not affected in *syp-4(tm2713)* mutants (Figure 4A–4D and data not shown), suggesting that SYP-4 acts downstream of axis morphogenesis. However, all three known central region components (SYP-1, SYP-2 and SYP-3) failed to localize to chromosomes in the *syp-4(tm2713)* mutants (Figure 4E–4L and data not shown). This interdependency of SYP-4 with the other SYP proteins is in agreement with a role for SYP-4 in central region assembly, as all SYP proteins exhibit similar interdependencies [2],[13],[14].

### The Stabilization of Homologous Pairing Interactions Requires SYP-4

**Figure 5:**
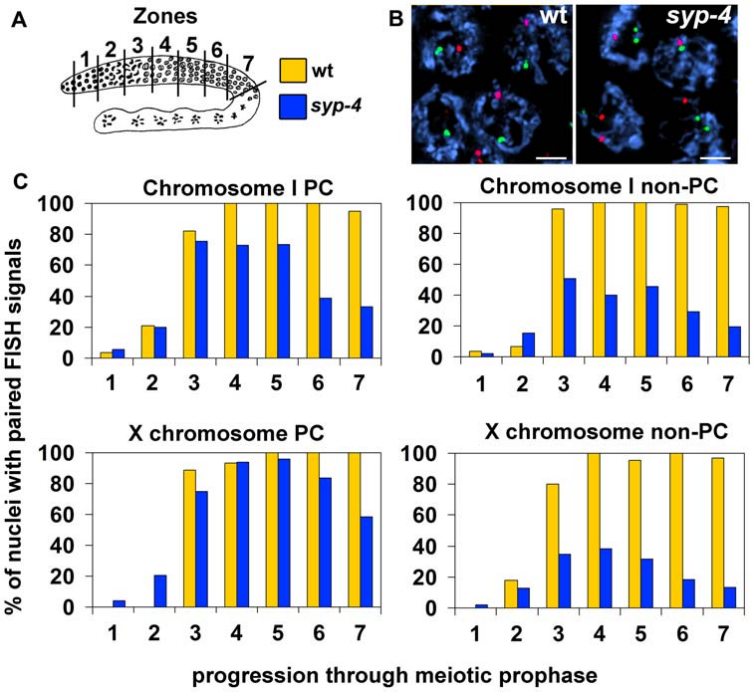
SYP-4 is required for the stabilization of homologous pairing interactions. (A) Diagram of a *C. elegans* germline indicating the position of the zones scored in the time-course analysis of homologous pairing. The color codes indicate the genotypes examined in (C). (B) High magnification images of pachytene nuclei from wild type and *syp-4* mutants stained with DAPI and hybridized with FISH probes recognizing either the PC end or the non-PC end of chromosome I (green and red, respectively). FISH signals are frequently unpaired in *syp-4* mutants. Bars, 2 µm. (C) Graphs depicting the percentage of nuclei carrying paired homologous chromosomes (y-axis) within each zone along the germline (x-axis). The regions being recognized by each of the FISH probes are indicated above the graphs.

In *C. elegans*, synapsis is crucial for the stabilization of chromosome paring interactions [2],[13],[14]. Since our immunolocalization studies place SYP-4 as a central region protein, we used fluorescence in situ hybridization (FISH) to monitor its role in chromosome pairing throughout prophase. We divided gonads from wild type and *syp-4(tm2713)* mutants into 7 zones and analyzed the percentage of nuclei carrying paired chromosomes in each one of these zones (Figure 5A). Specifically, we monitored pairing at opposite ends (the pairing center (PC) and non-PC ends) of chromosomes I and X. Levels of homologous pairing progressively increased in wild type nuclei as they entered meiosis in zones 2 to 3. In early pachytene (zone 4), chromosomes I and X were observed pairing with their homologous partners in approximately 100% of the nuclei examined and this level was maintained throughout late pachytene (zone 7) (Figure 5C). In contrast, although an increase in homologous pairing levels was detected in *syp-4(tm2713)* mutants initiating at the same time as in wild type, pairing levels failed to reach 100% and decreased as prophase progressed (Figure 5C, and Table S2, p<0.0001 for all loci in zones 6 and 7). This inability to stabilize pairing interactions was more pronounced for chromosome I than for the X chromosome, as exemplified by the observation of homologous pairing at the PC end of chromosome I in only 74% of the nuclei examined in zone 4, compared to 94% at the PC end of the X chromosome in the same zone (Figure 5C, p<0.0001). These differences probably reflect the yet unexplained propensity of the X chromosomes to pair more efficiently compared to the autosomes when SC formation is impaired, as seen in other mutants in *C. elegans* [35]–[37]. In addition, significantly higher levels of pairing were observed in the PC regions compared to the non-PC regions (Figure 5C). Specifically, pairing levels were 25% and 56% higher at the PC end compared to the non-PC end of chromosomes I and X, respectively (for Chromosome I, zone 3, p = 0.0061; for the X chromosome, zone 4, p<0.0001). The higher levels of pairing observed at the PC ends most likely stem from the fact that the initiation of homologous pairing events occurs at the PCs in a SYP-independent manner [2],[13],[14],[38]. Taken together, our observations indicate that unlike axis-associated proteins, SYP-4 is dispensable for the initiation of paring interactions, but as meiosis progresses it is crucial for the stabilization of pairing interactions. Our studies, therefore, further support a role for SYP-4 as a central region component of the SC.

### Normal Progression of Meiotic Recombination Requires SYP-4

**Figure 6:**
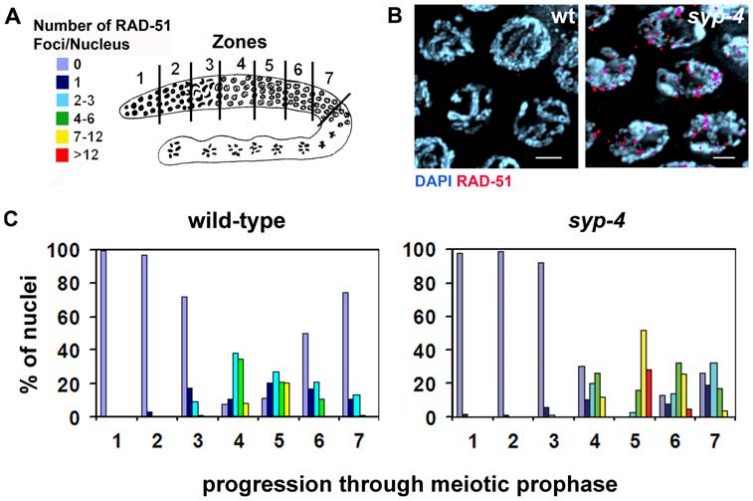
Progression of meiotic recombination is impaired in *syp-4* mutants. (A) Diagram of a *C. elegans* germline depicting the seven zones throughout which RAD-51 foci were scored for all nuclei. Levels of RAD-51 foci are indicated by the color code. (B) High magnification images of pachytene nuclei in wild type and *syp-4* mutants stained with DAPI and RAD-51. Elevated levels of RAD-51 foci are observed on pachytene nuclei in *syp-4* mutants. Bars, 2 µm. (C) Histograms depict the quantitation of RAD-51 foci in wild type and *syp-4* mutant germlines. The percentage of nuclei observed for each category indicated by the color code (y-axis), are depicted for each zone along the germline (x-axis).

Interhomolog recombination resulting in crossover events is dependent on chromosome synapsis [2],[13],[14]. Therefore, we examined the progression of meiotic recombination in the *syp-4(tm2713)* mutants by immunostaining whole mounted germlines with an anti-RAD-51 antibody (RAD-51 is required for strand invasion/exchange during double-strand break repair; [39]). Specifically, wild type and *syp-4(tm2713)* mutant germlines were divided into 7 zones and levels of RAD-51 foci/nucleus were quantitated for all nuclei in each zone (Figure 6A). In wild type gonads, levels of RAD-51 foci started to increase as nuclei entered into meiotic prophase and peaked in early to mid pachytene (Figure 6C, zone 5, 3.7 foci/nucleus), after which they gradually declined (Figure 6C, zone 7, 0.5 foci/nucleus). As nuclei exited pachytene, RAD-51 foci were no longer observed. In *syp-4(tm2713)* mutants, the increase in the levels of RAD-51 foci was first observed with a similar timing to wild-type, suggesting that the initiation of meiotic recombination is not dependent on SYP-4 (Figure 6C). However, levels of RAD-51 foci were significantly higher in mid-pachytene in *syp-4(tm2713)* mutants compared to wild type (Figure 6B and 6C, zone 5, 10.2 foci/nucleus; p<0.0001, two-tailed Mann-Whitney test, 95% C.I.) and remained elevated up to late pachytene (Figure 6C, zone 7, 2.1 foci/nucleus; p<0.0001, two-tailed Mann-Whitney test, 95% C.I.). The defect in DSB repair progression observed in *syp-4(tm2713)* mutants probably stems from the lack of chromosome synapsis and therefore a lack of close and stable proximity to a homologous template for repair. However, DSB repair is eventually accomplished in *syp-4(tm2713)* mutants, as RAD-51 foci were absent in diplotene nuclei and chromosome fragments were not apparent in oocytes at diakinesis (Figure 2H). This delayed repair may proceed in part through recombination with the sister chromatid, as previously demonstrated for *syp-2* and *syp-3* mutants [13],[40].

Unrepaired meiotic DSBs that persist until late pachytene may activate a DNA damage checkpoint, resulting in an increase in germ cell apoptosis at that stage [41]. Therefore, we examined the levels of germ cell apoptosis in *syp-4(tm2713)* mutants compared to wild type, *spo-11* mutants, that lack DSB formation, and *syp-3* null mutants, which have significantly impaired DSB repair (Table 1). *syp-4(tm2713)* mutants (n = 57) showed a significant increase in the levels of apoptosis compared to wild type (n = 42) and *spo-11*(n = 17) worms (p<0.0001 for both pairwise combinations, two-tailed Mann-Whitney test, 95% C.I.), but did not differ significantly from *syp-3(ok758)* (n = 22) (p = 0.8471). The elevated germ cell apoptosis levels are dependent on meiotically induced DSBs, as in the absence of *spo-11*, *syp-4(RNAi)* failed to increase apoptosis to the levels observed for *syp-4(RNAi)* in the *spo-11/nT1* heterozygous background. Taken together, this analysis suggests that the defects in DSB repair progression observed by monitoring the levels of RAD-51 foci throughout prophase, are sufficient to activate a DNA damage checkpoint response, and that such a checkpoint is intact in the *syp-4(tm2713)* mutants.

**Table 1:** Germ cell apoptosis is elevated in *syp-4* mutants.

Genotype	n	Mean Number of Germ Cell Corpses +/- Standard Error
wild type	42	2.49+/−0.26
*spo-11/spo-11*	17	1.95+/−0.37
*spo-11/nT1* a	20	1.6+/−0.39
*spo-11/spo-11* a	19	2.26+/−0.47
*syp-4(tm2713)/syp-4(tm2713)*	57	7.89+/−0.83
*syp-3(ok748)/syp-3(ok748)*	22	8.29+/−0.35
*syp-4(RNAi);spo-11/nT1*	51	5.04+/−0.47
*syp-4(RNAi);spo-11/spo-11*	58	3.47+/−0.34

acontrol RNAi with empty pL4440 vector. Germ cell corpses were scored in adult hermaphrodites 20 hours post-L4 as in [59]. Statistical comparisons between genotypes were conducted using the two-tailed Mann-Whitney test. *syp-4(tm2713)* (n = 57; where n = number of gonad arms scored/genotype) differed significantly from wild type (n = 42) (p<0.0001), and from *spo-11*(n = 17) (p<0.0001), but did not differ significantly from *syp-3(ok758)* (n = 22) (p = 0.8471). *syp-4(RNAi);spo-11/nT1* (n = 51) differed significantly from *syp-4(RNAi);spo-11/spo-11* (n = 58) (p = 0.0122), *spo-11/nT1*(n = 20) (p<0.0001), and *spo-11/spo-11*(n = 17) (p = 0.0016). *syp-4(RNAi);spo-11/spo-11* and *spo-11/spo-11*(n = 17) did not differ significantly from each other (p = 0.0905).

To examine whether SYP-4 is required for crossover recombination we measured crossover frequencies in *syp-4(tm2713)* mutants for intervals spanning ∼80% of the X chromosome (Table 2) and ∼70% of chromosome V (Table S3), utilizing genetic markers and single-nucleotide polymorphism (SNP) markers, respectively. As expected, given the lack of chromosome synapsis, the lack of chiasmata, and the defects observed with the progression of meiotic recombination, crossover levels were significantly reduced for the genetic intervals examined on both chromosomes in the *syp-4(tm2713)* mutants compared to wild type (p<0.0001, respectively, by the two-tailed Fisher's Exact Test, 95% C.I.) (Table 2, Table S3). Altogether, these results suggest that the crucial role that SYP-4 plays in chromosome synapsis is essential for the normal progression of meiotic recombination and crossover formation.

**Table 2:** Crossover recombination is reduced on the X chromosome in *syp-4* mutants.

Genotype	Recombinant progeny	Total No. of progeny scored	Map distance (cM)
*+/syp-4; dyp-3 unc-3/++*	656 hermaphrodites	2288 hermaphrodites	34.7
	0 males	1 male	
*syp-4/syp-4; dyp-3 unc-3/++*	2 hermaphrodites	323 hermaphrodites	0.6
	1 male	154 males	

Recombination analysis was performed as in [59].

## Discussion

### SYP-4 Is a Component of the Central Region of the SC in *C. elegans*

Here we report the first example of the identification of a novel structural protein of the SC through the yeast two-hybrid approach. We succeeded in uncovering SYP-4 through its physical interaction with SYP-3. We provide multiple lines of evidence suggesting that SYP-4 is a novel structural component of the SC participating in central region formation. The localization pattern of SYP-4 is distinct from that of meiosis-specific cohesin or lateral element components. Specifically, SYP-4 is only observed associating onto chromosomes upon entrance into meiosis and not earlier as observed for cohesin. Moreover, in contrast to axis-associated components, SYP-4 remains localized only to the short axes, instead of both long and short axes, of the bivalents following chromosome remodeling in late pachytene, and SYP-4 is no longer chromosome-associated by the end of diakinesis. Furthermore, analysis of the *syp-4(tm2144)* mutants supports a role for SYP-4 downstream of axis formation. First, SYP-4 localization to chromosomes depends on axis-associated proteins and is absent from unsynapsed chromosomes (*him-8* mutants), while axis-associated proteins still localize to the unsynapsed chromosomes of the *syp-4(tm2144)* or *him-8* mutants. Second, our analysis of homologous pairing levels in the *syp-4(tm2144)* mutants clearly points to SYP-4 acting downstream of the establishment of pairing, which is dependent on axis-associated proteins. Third, *syp-4(tm2713)* mutants exhibit several phenotypes observed in null mutants for central region components such as the extended transition zone morphology and the accumulation of high levels of RAD-51 foci accompanied by increased apoptosis in late pachytene nuclei [2],[13],[14]. These phenotypes are clearly distinct from those of mutants in axis-associated proteins, which show a shortened transition zone and low levels of RAD-51 foci [33],[34]. Altogether, these data render strong support for a role of SYP-4 as a central region protein of the SC, and point to its crucial function in promoting stable interactions between homologous chromosomes leading to crossover formation.

### SYP-4 and SYP-3 May Form a Module of the Central Region of the SC

The fact that SYP-4 is interdependent with SYP-1, SYP-2 and SYP-3, suggests that these proteins act in a complex. This is further supported by our identification of SYP-4 via a yeast two-hybrid interaction with SYP-3. Our yeast two-hybrid analysis suggests that SYP-3 may interact with SYP-4 through its N-terminal domain, since a C-terminal truncated SYP-3 can still interact with SYP-4, but a N-terminal truncation cannot. We were unable to detect any interaction between SYP-3 and any truncated version of SYP-4, implying that the full length of the protein is likely required for this interaction. In addition, using the yeast two-hybrid system, we did not observe an interaction between SYP-4 and HIM-3, an axis-associated component and yeast Hop1 homolog [27], HTP-3, an axis-associated component and HIM-3 paralog proposed to link DSB formation with homologous pairing and synapsis [34],[42], HTP-1, a HIM-3 paralog implicated in coordinating the establishment of pairing and synapsis in early prophase and involved in the crossover-dependent chromosome remodeling process observed in late prophase [36],[37], and ZHP-3, the ortholog of budding yeast Zip3 proposed to couple recombination with SC morphogenesis in *C. elegans* [43] (data not shown). Furthermore, an interaction between SYP-4 and either SYP-1 or SYP-2 was not detected when assessing for these pairwise interactions via the yeast two-hybrid system. These interactions were also not detected when we used SYP-1 and SYP-2 as baits in yeast two-hybrid screens of both cDNA and ORFeome libraries [44], nor reported by any other large genomic screen published in the literature. Therefore, these observations suggest that SYP-4 might interact exclusively with SYP-3. However, taking the limitations of the yeast two-hybrid system into account, we cannot exclude the possibility that other interactions were missed by this approach and may be detected using other experimental techniques. In addition, pull-down assays revealed that SYP-1 interacts with SYP-2 (K.S-P. and M.P.C unpublished results), but we were unable to test other pairwise combinations by this approach due to technical limitations. Taken together, these observations lead us to hypothesize that the central region of the SC in *C. elegans* may be comprised of at least two modules: one consisting of SYP-1 and SYP-2, and the other formed by SYP-3 and SYP-4. However, given that all SYP proteins are interdependent [13],[14, this study], these two sub-complexes must be either directly or indirectly interconnected, forming the higher-order structure of the central region of the SC. Alternatively, it is possible that all proteins assemble into a single complex lacking any sub-modules. Future studies are required to conclude which of these models accurately describes the structure of the SC in *C. elegans*.

### Possible Roles for the Multiple Central Region Proteins in *C. elegans*

Studies in various model organisms are revealing the identity of the proteins forming the central region of the SC: C(3)G and CONA in *Drosophila* [11],[12], Zip1 in *S. cerevisiae* [10], SYCP1, SYCE1, SYCE2 and TEX12 in mouse [15],[16],[18],[24], and the duplicated ZYP1a and ZYP1b proteins in *Arabidopsis* [45]. In mice, specific functions have been assigned to each one of four known CR proteins based on their distinct mutant phenotypes. A null *Sycp1* mutant results in a lack of CE formation and in the absence of any recruitment of CR proteins onto chromosomes, whereas foci for some of the CR components are still observed in *Syce2, Syce1* and *Tex12* mutants [17],[24],[46]. This is further supported by the observation of partial CR structures by EM in the case of *Syce2* and *Tex12* mutants [17],[24],[46]. In addition, SYCP1 (993 amino acids) is three to four times larger than any other CR protein. Thus, these studies, complemented by a detailed immuno-EM analysis, have led to the conclusion that SYCP1 acts as a transverse filament, contributing to most of the width of the CR structure, while SYCE1, SYCE2 and TEX12 are central element proteins that play an essential role in the assembly of the SC, but do not contribute much to the width of the structure. The *Drosophila* CR proteins, C(3)G and CONA, are structurally distinct from each other. Specifically, CONA (207 amino acids) is almost a quarter of the size of C(3)G and lacks any coiled-coil domain. Nevertheless, both *c(3)G* and *cona* mutants exhibit similar phenotypes [11], [12]. Thus, extrapolating from the mouse data, C(3)G may be the fly TF protein, while CONA may be a non-TF CR protein. The studies of the SC structure in *C. elegans* result in a more complex picture in which it is still hard to distinguish which of the SYP proteins act as bona fide TF proteins and which, if any, are central element-like proteins. In *C. elegans*, the total length of the coiled coils is predicted to be higher in SYP-1 (34.16 nm) compared to SYP-2 (5.05 nm), SYP-3 (13.51 nm) and SYP-4 (12.92 nm). Interestingly, the total length of the coiled coils in SYP-1 is smaller than that predicted for C(3)G in *D. melanogaster* (67.86 nm), Zip1 in *S. cerevisiae* (68.16 nm) and SYCP1 in *M. musculus* (87.76 nm) [12]. However, the width of the SC is conserved across most species (∼100 nm), including *C. elegans* [4],[35],[47],[48]. One possible scenario is that all the SYP proteins assemble into a single complex that can span the width of the SC. Therefore, the shorter lengths predicted for the coiled coils of each central region protein may be compensated by their additive value in *C. elegans*. This model suggests that in *C. elegans* multiple proteins may have evolved out of the need to conserve the width of the SC. An alternative, albeit not mutually exclusive model, is that in *C. elegans* different central region proteins play different roles in the 3-dimensional context of the SC, similarly to what is observed for central element proteins in mice. It is tempting to speculate that SYP-2 (213 amino acids) and SYP-3 (224 amino acids), the smaller proteins of the central region in *C. elegans*, which are similar in size to the mammalian central element proteins, take over a similar role in SC assembly. However, unlike what is observed in synapsis-defective mutants in mice, *syp* null mutants do not show a separation of function phenotype. This would be consistent with the notion that instead of each SYP protein loading individually onto chromosomes, the two SYP protein subunits (SYP-1/SYP-2 and SYP-3/SYP-4) preassemble into one complex and then load onto chromosomes as one unit composed of all four SYP proteins. Therefore, loss of either one of the two subunits will lead to defects in loading of the other SYP proteins, consequently leading to a complete perturbation of CR assembly. Either model would explain the interdependency between the various SYP proteins observed by our analysis, and both models predict that, albeit taking the yeast two-hybrid assay limitations into account, a yet non-identified additional component may exist linking SYP-1/SYP-2 and SYP-3/SYP-4.

In summary, our studies have identified SYP-4 as a novel component of the SC and revealed a key protein-protein interaction required between the central region proteins SYP-4 and SYP-3 to form the mature SC structure in *C. elegans*.

## Materials and Methods

### Genetics

All *C. elegans* strains were cultured at 20°C under standard conditions [49]. Bristol N2 worms were utilized as the wild type background, while Hawaiian CB4856 wild type worms were used for assessing recombination frequencies when utilizing single-nucleotide polymorphism (SNP) markers. The following mutations and chromosome rearrangements were used ([2], [13], [14], [27], [50]–[52]; this work):

LGI: *syp-3(ok758), hDf8, syp-4(tm2713), ccIs4251, hT2[bli-4(e937) qIs48] (I;III)*

LGIV: *him-3(gk149)*, *spo-11(ok79)*, *nT1[unc-?(n754) let-?(m435)] (IV;V)*

LGV: *syp-2(ok307), syp-1(me17)*

The *tm2713* allele was generated by the *C. elegans* National Bioresource Project in Japan. It contains a 213 base pair out-of-frame deletion including exon 2 and extending halfway into exon 3 of open reading frame H27M09.3.

### Characterization of Allele

Identical cytological defects to those observed in *syp-4(tm2713)* homozygotes were observed in *syp-4(RNAi)* worms, including an extended transition zone phenotype and up to 12 univalents at diakinesis. Moreover, *trans*-heterozygotes for *tm2713* and *hDf8*, a deficiency encompassing the *syp-4* locus, were indistinguishable from *tm2713* homozygotes, as determined by examining their DAPI-stained germlines and scoring for the embryonic lethality and the percent of males observed among their surviving progeny (98.6% embryonic lethality, p = 0.2713, and 29% male progeny, p = 0.6006, by the two-tailed Mann-Whitney test, 95% C.I.; n = 1598). Finally, SYP-4 signal was not detected upon immunostaining *syp-4* mutant germlines with an N-terminal anti-SYP-4 antibody. Taken together, these results suggest that *syp-4(tm2713)* is a null.

*tm2713* is a recessive *syp-4* allele. DAPI-stained germlines of *syp-4*/+ hermaphrodites were identical to wild type germlines. The levels of embryonic lethality and males observed among *syp-4*/+ progeny were not statistically significant when compared to those observed for wild type (3.9% embryonic lethality, p = 0.0789, and 0.08% male progeny, p = 0.6439, two-tailed Mann-Whitney test; n = 2521).

### Coiled-Coil Analysis

The presence of coiled-coil domains within CR proteins was predicted utilizing the COILS program [25]. This program was run using the MTIDK matrix with a 21-residue window and applying an unweighted scan. Protein regions were predicted to adopt a coiled-coil conformation if the amino acids within those regions had scores of 0.5 or higher. To estimate the physical length of the coiled-coil domain in nm, the number of amino acids identified by this analysis was multiplied by 0.1485 nm, the mean axial rise per residue in a coiled-coil [54].

### Yeast Two-Hybrid Screen

Full-length cDNAs of the *syp-1, syp-2* and *syp-3* open reading frames, as well as C- and N-terminal truncations that retain the coiled-coil domains, were amplified by PCR. The amplification was performed from a cDNA library generated from mixed-stage *C. elegans* using primers that contain Gateway compatible sequences and a gene specific sequence as in [55] and indicated in Table S1. Gateway cloning, cDNA and ORFeome library screening, and X-Gal and 3AT assays for examining yeast two-hybrid interactions were performed as in [56].

### RNA Interference

RNAi-mediated depletion of *syp-4* was performed at 20°C as described in [53], except that 1 mM IPTG was utilized. SYP-4 cDNA was cloned into the pL4440 feeding vector. Control RNAi was performed by feeding worms with HT115 bacteria carrying the empty pL4440 vector.

### Electron Microscopy

Wild type and *syp-4(tm2713)* adult hermaphrodites (20–24 hr post-L4) were prepared for high pressure freezing as described in [2]. 100 nm-thick longitudinal sections of three wild type worms and three *syp-4* mutant worms were examined for the presence of SC in nuclei at the late pachytene region. Distances between electron-dense chromatin patches arranged in parallel were measured and the presence of an SC was only scored positively when distances were within the range observed in wild type (90 nm–125 nm; [35]) for all points measured along a given pair of patches (between 1 to 4 points were measured for each pair). SC stretches were observed in 64% of the wild type nuclei in late pachytene (n = 70). In contrast, dispersed patches of electron dense chromatin, indicating a lack of SC, were observed in 96.4% of the *syp-4(tm2713)* nuclei at this stage (n = 83).

### FISH and Time Course Analysis of Chromosome Pairing

FISH probes were generated as in [21] from the following pooled cosmids obtained from the Sanger Center: D1037, ZC535, F21A9 (I, left); F14B11, F32A7 (I, right); F28C10, F57C12, F13C5, M6, M02A10, C02H7, T04G9, F25E2, C03F1, F56F10, ZC13 (X, left); T23E7, F20B4, F15G10, K09G11 (X, right). Cosmids were labeled with either fluorescein-12-dCTP (PerkinElmer) or Digoxigenin-11-dUTP (Roche). Homologous pairing was monitored quantitatively as in [57], with FISH signals considered paired when separated by ≤0.75 µm. The average number of nuclei scored per zone (n) from three gonads each for wild type and *syp-4 (tm2713)* are as follows: zone 1 (n = 53), zone 2 (n = 67), zone 3 (n = 88), zone 4 (n = 96), zone 5 (n = 104), zone 6 (n = 94), and zone 7 (n = 78).

### Antibody Preparation, DAPI Analysis, and Immunostaining

The rabbit α-SYP-4 N-terminal polyclonal antibody was generated using the following peptide antigen: MSFPTLQVRPNEKNPKVLRCHEFLRQS. Animals were immunized and bled by Sigma-Genosys, The Woodlands, TX. Affinity purification of this antibody was performed using SulfoLink® from Pierce following the manufacturers instructions.

DAPI staining, immunostaining and analysis of stained meiotic nuclei were performed as in [13]. Primary antibodies were used at the following dilutions: rabbit α-SYP-1 (1∶100), rabbit α-SYP-2 (1∶100), rabbit α-SYP-3 (1∶100), rabbit α-RAD-51 (1∶100), rabbit α-HIM-3 (1∶100), guinea pig α-HTP-3 (1∶500), and mouse α-REC-8 (1∶100). The secondary antibodies used were: Cy3 anti-rabbit, FITC anti-guinea pig and FITC anti-mouse (Jackson Immunochemicals), each at 1∶100.

The images were acquired using the DeltaVision wide-field fluorescence microscope system (Applied Precision) with Olympus 40×/1.35 and 100×/1.40 lenses. Optical sections were collected at 0.20 µm increments with a coolSNAP_HQ_ camera (Photometrics) and SoftWoRx 3.3.6 software (Applied Precision), and deconvolved using SoftWoRx 3.3.6 software. Images are projections halfway through 3D data stacks of whole nuclei (15 to 30 0.2 µm slices/stack), except for diakinesis images, which encompass entire nuclei, prepared using SoftWoRx 3.3.6 and SoftWoRx Explorer 1.3.0 software (Applied Precision).

### Time Course Analysis for RAD-51 Foci

Quantitation of RAD-51 foci was performed for all seven zones composing the germline as in [13]. The average number of nuclei scored per zone (n) from three gonads each for wild type and *syp-4(tm2713)* were: zone 1 (n = 172), zone 2 (n = 243), zone 3 (n = 238), zone 4 (n = 200), zone 5 (n = 166), zone 6 (n = 131) and zone 7 (n = 119).

### Determining Crossover Frequencies

Meiotic crossover recombination frequencies for chromosome V were assayed utilizing single-nucleotide polymorphism (SNP) markers, with the pkP5076 and snp_Y17D7B *Dra*I SNP primers as in [58]. *syp-4* homozygous cross-progeny were detected by mating to the *ccIs4251* strain as described in [42].

## Supporting Information

Figure S1 A yeast two-hybrid approach reveals that SYP-4 interacts with SYP-3 through its N-terminal domain. Yeast two-hybrid analysis using growth on 20 mM 3-AT plates to identify protein-protein interactions between the SYP proteins. Testing the interactions between SYP-1, SYP-2, and SYP-3 full length, N- (ΔN) and C-terminal (ΔC) truncations fused to the DNA binding domain (DB) of GAL4, and SYP-4 full length fused to the activation domain (AD) of GAL4, revealed an interaction between SYP-4 and SYP-3. Numbers 1 to 5 represent standard controls: 1, DB and AD without any fusion; 2, DB-pRB and AD-E2F1; 3, DB-Fos and AD-Jun; 4, Gal4p and AD; and 5, DB-DP and AD-E2F1.
(0.68 MB TIF)

Figure S2 TEM analysis reveals a lack of SC formation in *syp-4* mutants. TEM images of equatorial sections of late pachytene nuclei from wild type (left) and *syp-4* (right) germlines. The nucleolus, a large dark body slightly off-center, is surrounded by electron-dense patches of chromatin, which are aligned in wild type nuclei, but are randomly positioned in *syp-4* mutants where SC structures are absent. Bars, 500 nm.
(4.45 MB TIF)

Table S1 Primers used for the yeast two-hybrid experiments.
(0.02 MB DOC)

Table S2 Statistical analysis for FISH data.
(0.04 MB DOC)

Table S3 Crossover recombination is reduced on chromosome V in *syp-4* mutants.
(0.02 MB DOC)
