# Supplementary figures and images for: A Yeast Two-Hybrid Screen for SYP-3 Interactors Identifies SYP-4, a Component Required for Synaptonemal Complex Assembly and Chiasma Formation in Caenorhabditis elegans Meiosis
Source: PLoS Genet. 2009 Oct 2;5(10):e1000669. doi: 10.1371/journal.pgen.1000669 (PMC2742731; doi:10.1371/journal.pgen.1000669)

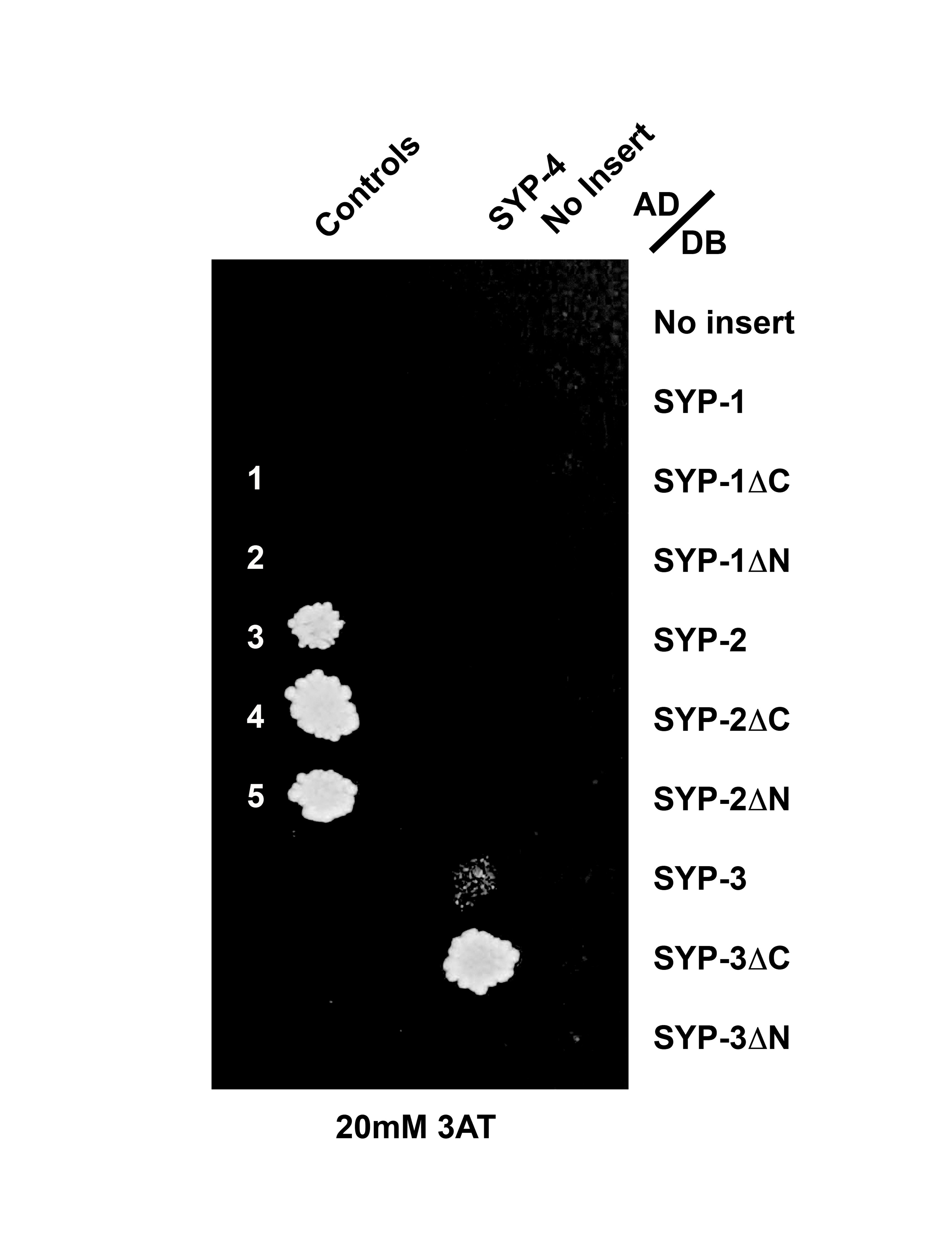

Supplement: Figure S1 — A yeast two-hybrid approach reveals that SYP-4 interacts with SYP-3 through its N-terminal domain. Yeast two-hybrid analysis using growth on 20 mM 3-AT plates to identify protein-protein interactions between the SYP proteins. Testing the interactions between SYP-1, SYP-2, and SYP-3 full length, N- (ΔN) and C-terminal (ΔC) truncations fused to the DNA binding domain (DB) of GAL4, and SYP-4 full length fused to the activation domain (AD) of GAL4, revealed an interaction between SYP-4 and SYP-3. Numbers 1 to 5 represent standard controls: 1, DB and AD without any fusion; 2, DB-pRB and AD-E2F1; 3, DB-Fos and AD-Jun; 4, Gal4p and AD; and 5, DB-DP and AD-E2F1. (0.68 MB TIF) [file pgen.1000669.s001.tif]

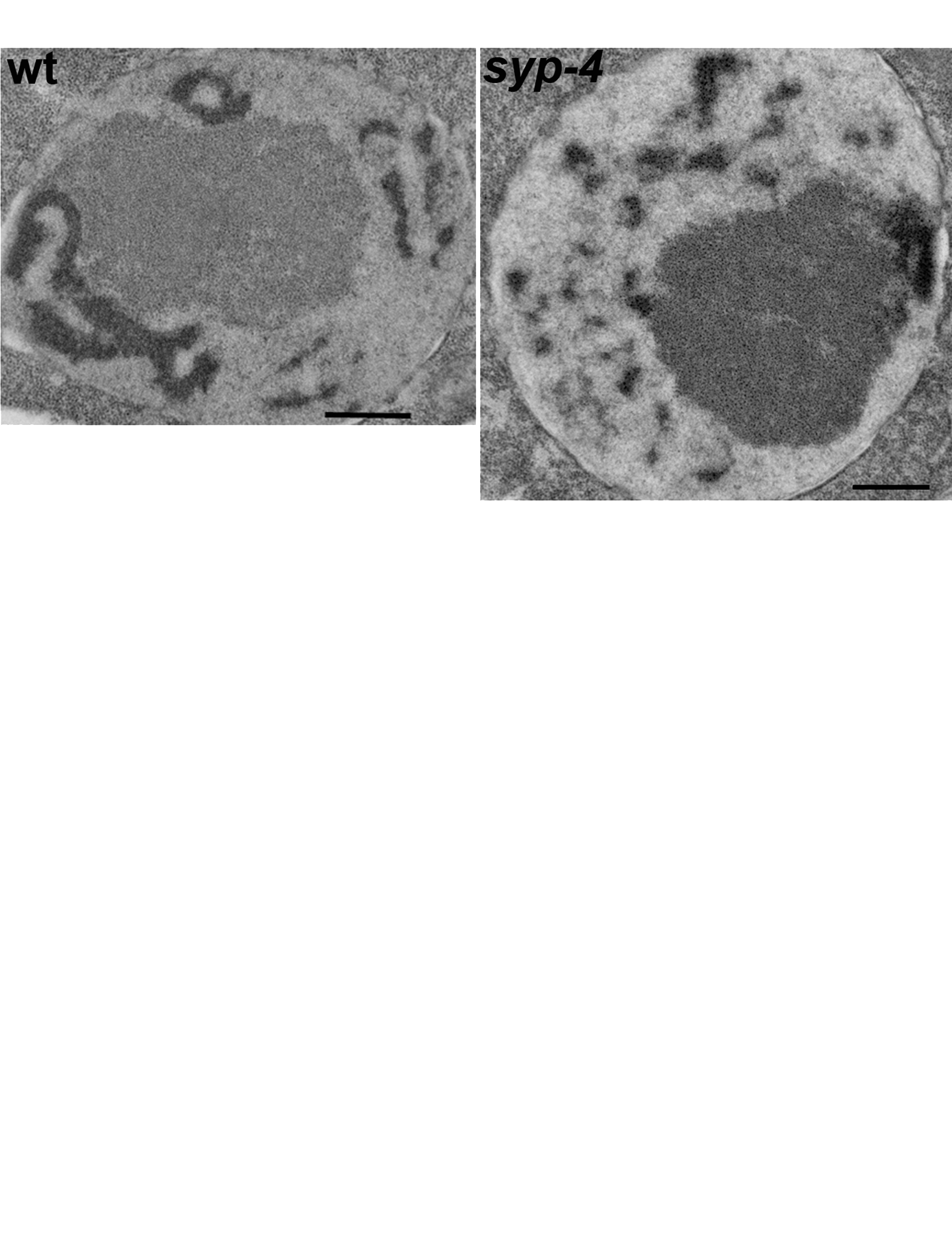

Supplement: Figure S2 — TEM analysis reveals a lack of SC formation in syp-4 mutants. TEM images of equatorial sections of late pachytene nuclei from wild type (left) and syp-4 (right) germlines. The nucleolus, a large dark body slightly off-center, is surrounded by electron-dense patches of chromatin, which are aligned in wild type nuclei, but are randomly positioned in syp-4 mutants where SC structures are absent. Bars, 500 nm. (4.45 MB TIF) [file pgen.1000669.s002.tif]
