# Supplementary material for: A Yeast Two-Hybrid Screen for SYP-3 Interactors Identifies SYP-4, a Component Required for Synaptonemal Complex Assembly and Chiasma Formation in Caenorhabditis elegans Meiosis
Source: PLoS Genet. 2009 Oct 2;5(10):e1000669. doi: 10.1371/journal.pgen.1000669 (PMC2742731; doi:10.1371/journal.pgen.1000669)
Supplement: Table S1 — Primers used for the yeast two-hybrid experiments. (0.02 MB DOC) [file pgen.1000669.s003.doc]

**Table S1. Primers used for the yeast two-hybrid experiments.**

| **Primer name** | **Primer sequence** |
| --- | --- |
| SYP-1-full length-F | CATGGATAACTTCACAATTTGGGT |
| SYP-1-full length-R | GTATTTCTTCCCTCCTCTCTTT |
| SYP-1-ΔC-F | CATGGATAACTTCACAATTTGGGT |
| SYP-1-ΔC-R | AGCACGTTGTTCTTTAATTTGATT |
| SYP-1-ΔN*-*F | CGAGCAAGATAAAGGCGAGCA |
| SYP-1-ΔN*-*R | GTATTTCTTCCCTCCTCTCTTT |
| SYP-2-full length-F | CATGAATTCTGCCCATCGATCT |
| SYP-2-full length-R | GTATAACTTGTCAGCCCAC |
| SYP-2-ΔC-F | CATGAATTCTGCCCATCGATCT |
| SYP-2-ΔC-R | TCTGGCGTCAACGCGATTT |
| SYP-2-ΔN-F | CACACAAGATCTGACAATGGAGCTGG |
| SYP-2-ΔN-R | GTATAACTTGTCAGCCCAC |
| SYP-3-full length-F | ATGGTGACCGATTCAAAATTT |
| SYP-3-full length-R | TCATGTAGAAAGTCGGGCT |
| SYP-3-ΔC-F | ATGGTGACCGATTCAAAATTT |
| SYP-3-ΔC-R | ACGGAGAGAAGGAATCATTTCA |
| SYP-3-ΔN-F | GAAGACAGGGTTAAATGTGCTGCTCAA |
| SYP-3-ΔN-R | TCATGTAGAAAGTCGGGCT |
