# Supplementary material for: A Yeast Two-Hybrid Screen for SYP-3 Interactors Identifies SYP-4, a Component Required for Synaptonemal Complex Assembly and Chiasma Formation in Caenorhabditis elegans Meiosis
Source: PLoS Genet. 2009 Oct 2;5(10):e1000669. doi: 10.1371/journal.pgen.1000669 (PMC2742731; doi:10.1371/journal.pgen.1000669)
Supplement: Table S2 — Statistical analysis for FISH data. (0.04 MB DOC) [file pgen.1000669.s004.doc]

**Table S2.** **Statistical analysis for FISH data.**

|  | | Z 1 | | Z2 | | Z3 | | Z4 | | Z5 | | Z6 | | Z7 | |
| --- | --- | --- | --- | --- | --- | --- | --- | --- | --- | --- | --- | --- | --- | --- | --- |
| Genotype | Probe | P | UP | P | UP | P | UP | P | UP | P | UP | P | UP | P | UP |
| wild type | I-PC | 2 | 54 | 16 | 61 | 79 | 17 | 95 | 0 | 101 | 0 | 111 | 0 | 74 | 4 |
| *syp-4* |  | 3 | 49 | 14 | 57 | 49 | 16 | 60 | 22 | 66 | 24 | 24 | 38 | 17 | 34 |
| *p-V*alue |  | 0.6703 | | 1 | | 0.3229 | | <0.0001* | | <0.0001* | | <0.0001* | | <0.0001* | |
| wild type | I-NPC | 2 | 54 | 5 | 72 | 92 | 4 | 95 | 0 | 101 | 0 | 110 | 1 | 76 | 2 |
| *syp-4* |  | 1 | 51 | 11 | 60 | 33 | 32 | 33 | 49 | 41 | 49 | 18 | 44 | 10 | 41 |
| *p-V*alue |  | 1 | | 0.1112 | | <0.0001* | | <0.0001* | | <0.0001* | | <0.0001* | | <0.0001* | |
| wild type | X-PC | 1 | 57 | 1 | 55 | 98 | 12 | 86 | 3 | 101 | 0 | 95 | 1 | 99 | 0 |
| *syp-4* |  | 2 | 46 | 13 | 50 | 62 | 21 | 110 | 7 | 119 | 5 | 91 | 18 | 49 | 35 |
| *p-V*alue |  | 0.5887 | | 0.0013* | | 0.0116* | | 0.5197 | | 0.0662 | | <0.0001* | | <0.0001* | |
| wild type | X-NPC | 2 | 56 | 5 | 51 | 90 | 20 | 87 | 2 | 99 | 2 | 88 | 7 | 96 | 3 |
| *syp-4* |  | 1 | 47 | 8 | 55 | 29 | 54 | 45 | 72 | 39 | 85 | 20 | 89 | 11 | 73 |
| *p-V*alue |  | 1 | | 0.5682 | | <0.0001* | | <0.0001* | | <0.0001* | | <0.0001* | | <0.0001* | |

The significance of the pairing levels was examined by the Fisher’s Exact Test (two-tailed *p* value, 95% confidence interval) using the InStat software (Graphpad). Z1 to Z7 correspond to zones 1 through 7 indicated in Figure 5. P= Number of nuclei with paired FISH signals for indicated probe. UP= Number of nuclei with unpaired FISH signals for indicated probe; * statistically significant.
