# Supplementary material for: A Yeast Two-Hybrid Screen for SYP-3 Interactors Identifies SYP-4, a Component Required for Synaptonemal Complex Assembly and Chiasma Formation in Caenorhabditis elegans Meiosis
Source: PLoS Genet. 2009 Oct 2;5(10):e1000669. doi: 10.1371/journal.pgen.1000669 (PMC2742731; doi:10.1371/journal.pgen.1000669)
Supplement: Table S3 — Crossover recombination is reduced on chromosome V in syp-4 mutants. (0.02 MB DOC) [file pgen.1000669.s005.doc]

**Table S3.** **Crossover recombination is reduced on chromosome V in *syp-4* mutants.**

| **Genotype** | **No. of recombinants** | **Total No. of worms examined** | **Map distance (cM)** |
| --- | --- | --- | --- |
| *syp-4/+* | 33 | 95 | 34.7 |
| *syp-4/syp-4* | 2 | 58 | 3.4 |

*^a^*SNPs used for chromosome V: pkP5076 and snp_Y17D7B
